# Photon-counting CT-angiography in comparison to digital subtraction angiography for assessing intracranial aneurysms after coiling or clipping

**DOI:** 10.1007/s00234-025-03650-w

**Published:** 2025-06-02

**Authors:** Frederic De Beukelaer, Laura Wuyts, Sophie De Beukelaer, Steven Van Hedent, Omid Nikoubashman, Martin Wiesmann, Michael Veldeman, Rastislav Pjontek, Anke Höllig, Hani Ridwan, Charlotte Weyland

**Affiliations:** 1 Department of Neuroradiology, University Hospital Aachen, Aachen, Germany; 2 Department of Radiology, AZ Sint-Lucas, Ghent, Belgium; 3 Department of Neurology, University Hospital Bern, Bern, Switzerland; 4 Department of Neurosurgery, University Hospital Aachen, Aachen, Germany

**Keywords:** Photon Counting CT, CT angiography, Aneurysm treatment, Coil-embolization, Microsurgial clipping

## Abstract

**Purpose:**

To evaluate the potential of Photon-Counting Detector CT Angiography (PCD-CTA) for the post-interventional assessment of intracranial aneurysms treated with coil-embolization or clipping, compared to digital subtraction angiography (DSA).

**Methods:**

Retrospective analysis of consecutive patients treated with coils or clips between April 2023 and May 2024, who underwent PCD-CTA and, if necessary, DSA as part of their clinical routine. Polyenergetic images and spectral reconstructions were performed at different kiloelectron volt (keV) levels (40, 80 and 120) and with reconstruction kernels: Quantitative (Qr56 and Qr72) and Head vessel (Hv56 and Hv72), both with and without iterative metal artifact reduction (iMAR). Three independent readers assessed image quality using a 5-point Likert scale and region of interest analysis. A blinded, independent reading was performed to determine the presence of aneurysm remnants and intracranial vessel stenosis in the parent vessel.

**Results:**

A total of 21 patients (mean age 58 ± 14 years; range 36–74; 18 women) with intracranial, saccular aneurysms treated with either clipping (17/21) or coiling (4/21) were included. Reconstructions using smooth kernels (Hv56, Qr56) at a low keV level (40 keV) yielded increased signal- and contrast-to-noise ratios compared to sharper kernels (Hv72, Qr72) and higher keV levels (80 and 120 keV) (*p* < 0.001). Unexpectedly, reconstructions with iMAR negatively impacted evaluation, with only 6/21 diagnostic images at the clip site. The sensitivity of PCD-CTA for detecting aneurysm remnants was 100% (7 of 7 aneurysm clip/coil site), while specificity was 89% for patients with clips (8/9). A 100% negative predictive value was observed for all readers regarding aneurysm remnants.

**Conclusion:**

Photon-Counting CT-Angiography demonstrated adequate diagnostic value in most patients with intracranial clips. However, while coil artifacts were reduced, spectral reconstructions and iMAR were not sufficient to fully minimize these artifacts.

**Supplementary Information:**

The online version contains supplementary material available at 10.1007/s00234-025-03650-w.

## Introduction

In clinical practice, imaging protocols to assess occlusion rate, aneurysm reperfusion, and parent vessel pathologies following clip or coil placement vary among centers. In general, MR angiography is the preferred imaging modality for patients treated with coil-embolization, whereas CT angiography or flat-panel CT are preferred for patients who have undergone microsurgical clipping [1]. 

Photon-Counting Detector CT Angiography (PCD-CTA) provides high spatial resolution, especially in ultra-high-resolution acquisition mode (UHR) with a minimum slice thickness of 0.2 mm [2]. It has shown its added value concerning diagnostic accuracy in multiple comparative studies [3–6]. 

Phantom studies reported promising results with reduced artifacts, especially using iterative metal artifact reduction (iMAR). Clip or coil related artifacts were found to be lowest at a high kiloelectron Volt (keV) level of 100. However, this comes at the expense of reduced contrast-to-noise ratio’s (CNR) for the parenchyma [7–12]. 

Intracranial clips and coils have not been assessed in vivo to compare PCD-CTA with other imaging modalities, especially DSA.

We aimed to assess the imaging post-processing possibilities of PCD-CTA for the evaluation of intracranial vessels after clip-placement or coil-embolization for the treatment of intracranial aneurysms. In this study, the best reconstruction kernels and keV-levels are defined to assess lumen visibility as well as to investigate the diagnostic accuracy of PCD-CTA, compared to DSA imaging.

## Methods

This study is a single-center, retrospective analysis of consecutive patients treated for intracranial aneurysms using either clip placement or coil embolization between April 2023 and May 2024. The local ethics committee approved the study (local registration number: EK 24–270). Patient consent was waived due to the retrospective nature of the study design. The study is reported in adherence to the STROBE criteria [13]. 

All patients were scanned with a dual-source photon-counting CT scanner (NAEOTOM Alpha, Siemens Healthineers^®^, Erlangen, Germany, software version syngo CT VB10) operated in ultra-high-resolution mode. The following acquisition parameters were used: tube voltage 140 or 120kvp, pitch 0.65, rotation time 0.5 s. CTA was performed after the administration of 80 ml iodinated contrast material (Ultravist-370 (generic name, iopromide; Bayer Healthcare, Berlin, Germany), injected through a 20-gauge intravenous antecubital vein catheter using a power injector. The flow rate 4 ml/s. Opacification of the common carotid artery was monitored using a bolus tracking technique. The start time of data acquisition was determined with a fixed delay of five seconds after the attenuation threshold was reached.

**Fig. 1:**
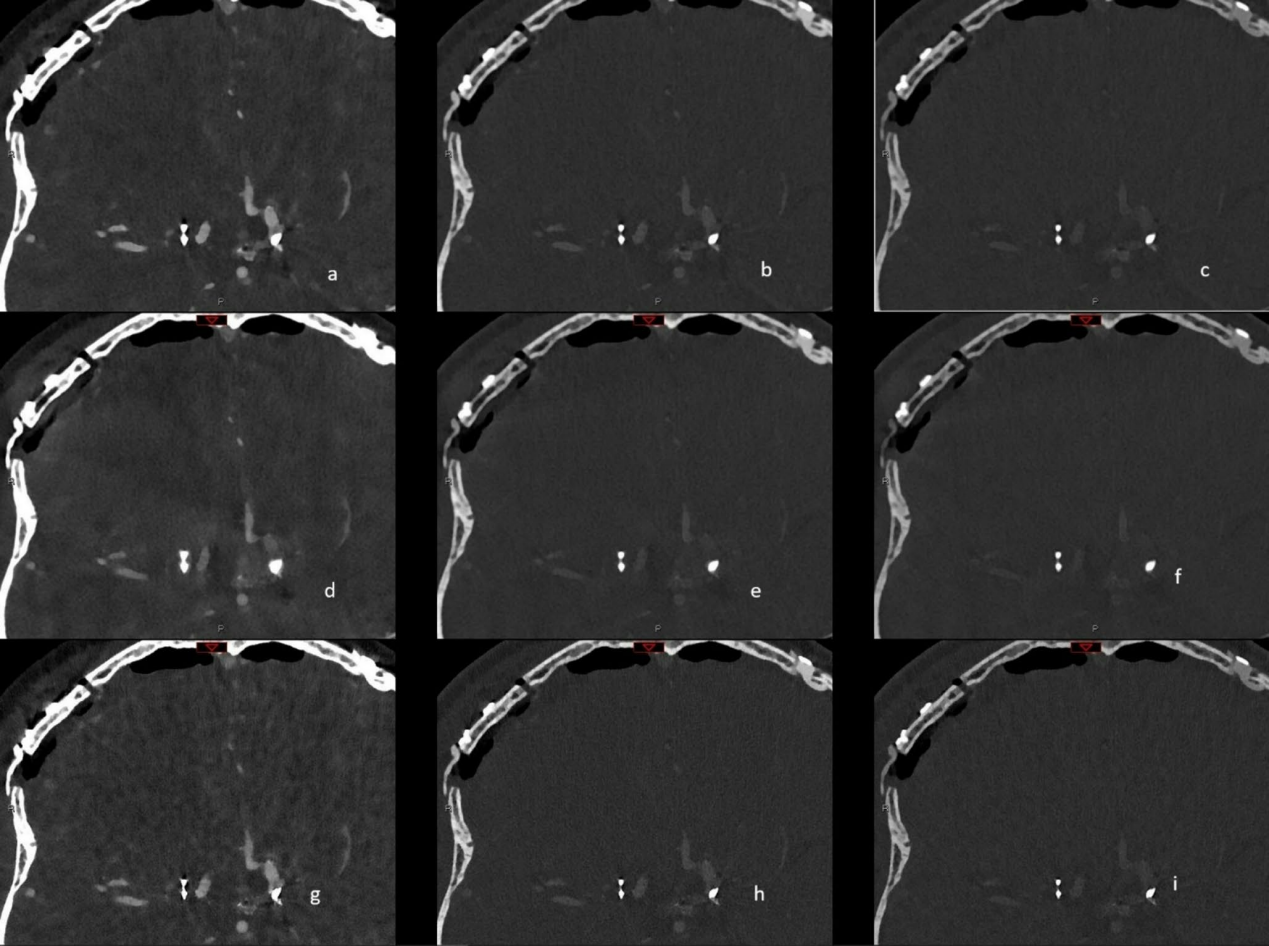
Illustration of reconstruction protocol for VMI with three different kernels and three keV levels. In the three rows, from left to right, the three keV levels (40, 80 and 120) are listed and in the three columns, from top to bottom Hv56 kernel without iMAR, Hv56 with iMAR and Hv 72. In detail: a = 40 keV, Hv 56; b = 80 keV, Hv 56; c = 120 keV, Hv 56; d = 40 keV, Hv56 with iMAR; e = 80 keV, Hv 56 with iMAR; f = 120 keV, Hv56 with iMAR; g = 40 keV, Hv 72; h = 80 keV; Hv72; i = 120 keV, Hv72. Abbreviations: Hv: Head vascular kernel, iMAR: iterative metal artefact reduction, keV: kiloelectron Volt, VMI: Virtual Monoenergetic Imaging

**Fig. 2:**
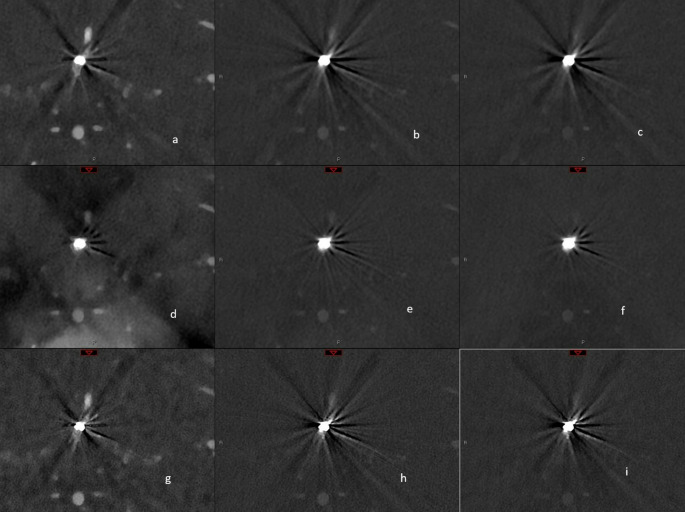
Illustration of the effect of coil artefacts in different reconstructions on the vascular representation in the immediate vicinity of the coiled aneurysm. In the three rows, from left to right, the three keV levels (40, 80 and 120) are listed and in the three columns, from top to bottom Hv56 kernel without iMAR, Hv56 with iMAR and Hv 72. In detail: a = 40 keV, Hv 56; b = 80 keV, Bv 56; c = 120 keV, Bv 56; d = 40 keV, Hv56 with iMAR; e = 80 keV, Hv 56 with iMAR; f = 120 keV, Hv56 with iMAR; g = 40 keV, Hv 72; h = 80 keV; Hv72; i = 120 keV, Hv72. Abbreviations: Hv: Head vascular kernel, iMAR: iterative metal artefact reduction, keV: kiloelectron Volt, VMI: Virtual Monoenergetic Imaging

In ten patients (6 patients with clips and 4 patients with coils) polyenergetic (PE) and iodine reconstructions (IOD) were performed with two different kernels (56 and 72), virtual monoenergetic imaging (VMI) reconstructions were performed using two kernel types (Quantitative (Qr) and Head vessel (Hv) kernel) with two kernel levels (56 and 72) and with three different keV levels (40, 80 and 120) as visualized in Figs. 1 and 2.

The chosen keV levels follow local expertise and existing recommendations for PCD-CT imaging. For example, virtual monoenergetic imaging (VMI) reconstructions at 40 keV using the kernel Bv 72 allow the visualization of in-stent vessel lumen (i.e. overcome the stent associated artefacts) in intracranial vessels [2–7]. 

Reconstructions with Qr56 or Hv56 were additionally processed using iMAR [14]. 

Polyenergetic reconstructions were reconstructed in 0.2 mm slice thickness and 0.1 mm slice increment.

Spectral reconstructions were reconstructed in 0.4 mm slice thickness and 0.2 mm slice increment.

The reconstructed matrix size was 1024 × 1024, and the field of view was adjusted for each patient to optimally image the parent vessel of the treated aneurysm.

After assessing these reconstructions, we analyzed the CT Angiography with the as superior evaluated reconstructions of 11 more patients.

### Subjective image quality assessment

Three radiologists with 10 years (blinded for review) and 11 years (blinded for review) of experience independently evaluated all images using the syngo.via workstation (version VB10 A, Siemens Healthineers) while remaining blinded to clinical data, as well as DSA imaging. Image quality was rated on a 5-point Likert scale (1 = “non-diagnostic,” 5 = “excellent”) based on lumen visibility of the parent vessel 1 cm before and at the aneurysm neck.

## Objective image quality assessments

Objective metrics, including contrast-to-noise ratios (CNR) and signal-to-noise ratios (SNR) are detailed in the online supplementary materials (Table S1). Each of the three radiologists independently placed four regions of interest (ROI) of identical diameter in each reconstruction in similar location: one in the parent vessel within 1 cm proximal to the coil or clip, one in the vessel adjacent to the clip or coil, one in the air above the calvarium, and one in the superficial temporal muscle. The mean Hounsfield units (HU) were recorded from these ROIs.

SNR and CNR were calculated as follows:

$$\begin{array}{c}\mathrm{SNR}={\mathrm{signal}}_{\mathrm{artery}}/\mathrm{standard}\;{\mathrm{deviation}}_{\mathrm{artery}}\\\mathrm{CNR}=\left({\mathrm{signal}}_{\mathrm{artery}}-{\mathrm{signal}}_{\mathrm{muscle}}\right)/\mathrm{standard}\;{\mathrm{deviation}}_{\mathrm{air}}\end{array}$$

### Assessment of aneurysm occlusion and patency of the parent vessel

Two readers (blinded for review) performed an independent assessment of aneurysm occlusion and the patency of the parent vessel. Segments scored as non-diagnostic due to impaired image quality on every reconstructed CT image were rated as potential sites for residual aneurysms or stenosis.

### DSA for the post-operative assessment of intracranial aneurysms

Cerebral DSA was performed on a biplane angiographic unit (Artis Zee SIEMENS, Erlangen, Germany) using standard techniques by board-certified neuroradiologists. At least two dedicated projections at high resolution of the in-stent vessel lumen were obtained after acquiring a three-dimensional (3D)-rotation angiography of the respective vessel territory to determine optimal projections. Two readers, blinded to the angiography reports, performed a dedicated study read.

DSA and Photon-Counting-CTA were performed during the same hospital admission, usually within two days (median, (0–5 (range)). Additionally, to the short interval between the evaluated modalities, there was no clinical or angiographic sign of parent vessel occlusion or cerebral vasospasm in between examinations.

### Radiation dose assessment

All participants’ radiation dose parameters, specifically the dose length product and the dose area product were extracted from the CT and DSA reports.

### Statistical analysis

IBM SPSS Statistics software (version 28.0) was used for statistical analysis.

Normality of distributions for descriptive analysis was assessed using the Shapiro-Wilk test. Quantitative variables were expressed as mean ± standard deviation (SD) or median and interquartile ranges (IQR). Likert scores were pooled across readers.

Friedman’s test was used to compare different kernels for each reconstruction type (IOD, PE, VMI), with pairwise comparisons. In case of the VMI, the three keV levels were compared for each kernel before using the test. Friedman’s test was used to compare the Likert values of the different optimized reconstructions (IOD, PE, VMI).

Interobserver agreement was expressed in Cohen’s kappa (κ) value and interpreted as follows: ≤ 0.20 as none, 0.21–0.40 as fair, and 0.41–0.60 as moderate, 0.61–0.80 as substantial, and ≥ 0.81 as almost perfect agreement [9]. All p-values were corrected for multiple comparisons using the Bonferroni correction.

A 95% confidence interval range (CI) was calculated for all diagnostic accuracy tests, and a two-tailed *p*-value of < 0.05 was considered statistically significant [15]. 

Diagnostic performance of PCD-CTA compared with DSA was evaluated using the pooled readers’ assessment of the 2 vessel sites in 21 patients. Receiver operating characteristic curve analysis was performed to calculate the area under the curve. Non-parametric distribution assumptions were made for standard error approximation.

Sensitivity, specificity, positive predictive value and negative predictive value were calculated for detecting parent vessel stenosis of ≥ 50% or residual aneurysm. Results were visualized using ggplot2 and Likert packages within R Software (The R Project for Statistical Computing, r-project.org) [16, 17]. 

## Results

Patient characteristics are detailed in Table 1. A total of 21 patients (60 ± 8 years (36–75) mean patient age; 13 women) with either clips (17/21) or coils (4/21) for the treatment of intracranial, saccular aneurysms were analyzed. Eight patients presented with ruptured aneurysms. Almost all patients with clips (16/17) were treated with YASARGIL^®^ Aneurysm Clips, B. Braun AG, Aesculap Inc, Tuttlingen, Germany.

**Table 1:** Patient characteristics

Age (years)*		58 ± 14
Sex (female/male)		18/3
Aneurysm Locations	Internal cerebral artery	2 (10)
	Middle cerebral artery	12 (57)
	Anterior cerebral artery	1 (5)
	Posterior communicating artery	2 (10)
	Anterior communicating artery	3 (13)
	Basilar artery	1 (5)
Aneurysm size (mm)*		6,4 ± 2,1
DLP mGycm*		113 ± 23
DAP µGycm^2^ *		1539 ± 648

— Except where indicated, data are numbers of participants, with percentages in parentheses

Abbreviations: *DAP *Dose area product, *DLP *Dose length product, *mGycm *Milligray centimeter, *mGycm*^2 ^Milligray square centimeter, mm: millimeter; * Data are means ± standard deviations

### Subjective and objective evaluation of image quality

Of the 21 patients, 42 vessel segments were evaluated, in 3 reconstructions (IOD, PE, VMI).

Virtual monoenergetic imaging reconstructions (VMI) reconstructions showed a decrease in SNR and CNR across different kernel and keV levels for both kernel types (Qr and Hv).

1. In VMI, the lowest keV-level (40 keV) achieved the highest SNRs and CNRs, significantly outperforming the highest keV level (120 keV). For example, SNR for Hv 56 at 40 keV was higher than at 80 keV in the parent vessel (mean ± SD: 24.9 ± 13.4 vs. 10.6 ± 14.3, *p* = 0.01), as well as in the vessel at the clip site (median (IQR): 13.6 (15.2) vs. 6.8 (6.1), *p* < 0.001).
2. Smoother kernels (Hv56) resulted in higher SNRs and CNRs compared to harder kernels (Hv72) at 40 keV. As an example, SNR values for Hv56 and Hv72 kernels for 40 keV were in the parent vessel 32.4 ± 10.6 (mean ± SD) vs. 17.2 ± 7.1 (median (IQR), *p* < 0.001), in the vessel at the clip or coil site 17.6 ± 10.9 vs. 13.3 ± 7.1 (mean ± SD, *p* = 0.03).
3. Reconstructions without iMAR showed comparable SNR and CNR in the parent vessel but unexpectedly differed at the clip or coil site.

As an example, SNR values for Hv56 and Hv56 with iMAR for 40 keV were in the intracranial parent vessel 32,4 ± 10,6 (mean ± SD) vs. 25,4 ± 15,4 (mean ± SD, *p* = 0.119).

However, SNR in the vessel at the clip or coil site differed in favor for reconstructions without iMAR (17,6 ± 10,9 vs. 11,7 (20, 6): *p* = 0.03).

For iodine and polyenergetic reconstructions, smoother kernels consistently provided the highest SNR and CNR in the parent vessel and in the vessel at the clip or coil site.

As an example, SNR values for Hv56 and Hv72 kernels were in the intracranial parent vessel 14,6 ± 2,8 (mean ± SD) vs. 8,5 ± 2,7 (mean ± SD, *p* < 0.001), in the vessel at the clip or coil site 7,8 (8, 9) vs. 4,8 (7, 7) (median (IQR): *p* < 0.001).

### Qualitative analysis

For VMI, images reconstructed at 40 keV achieved the highest score on the 5-point Likert scale (*p* < 0.001). For the parent vessel images reconstructed with Hv56, Hv72, Qr56 and Qr72 at 40 keV with and without iMAR achieved comparable results (*p* = 0.12).

**Fig. 3:**
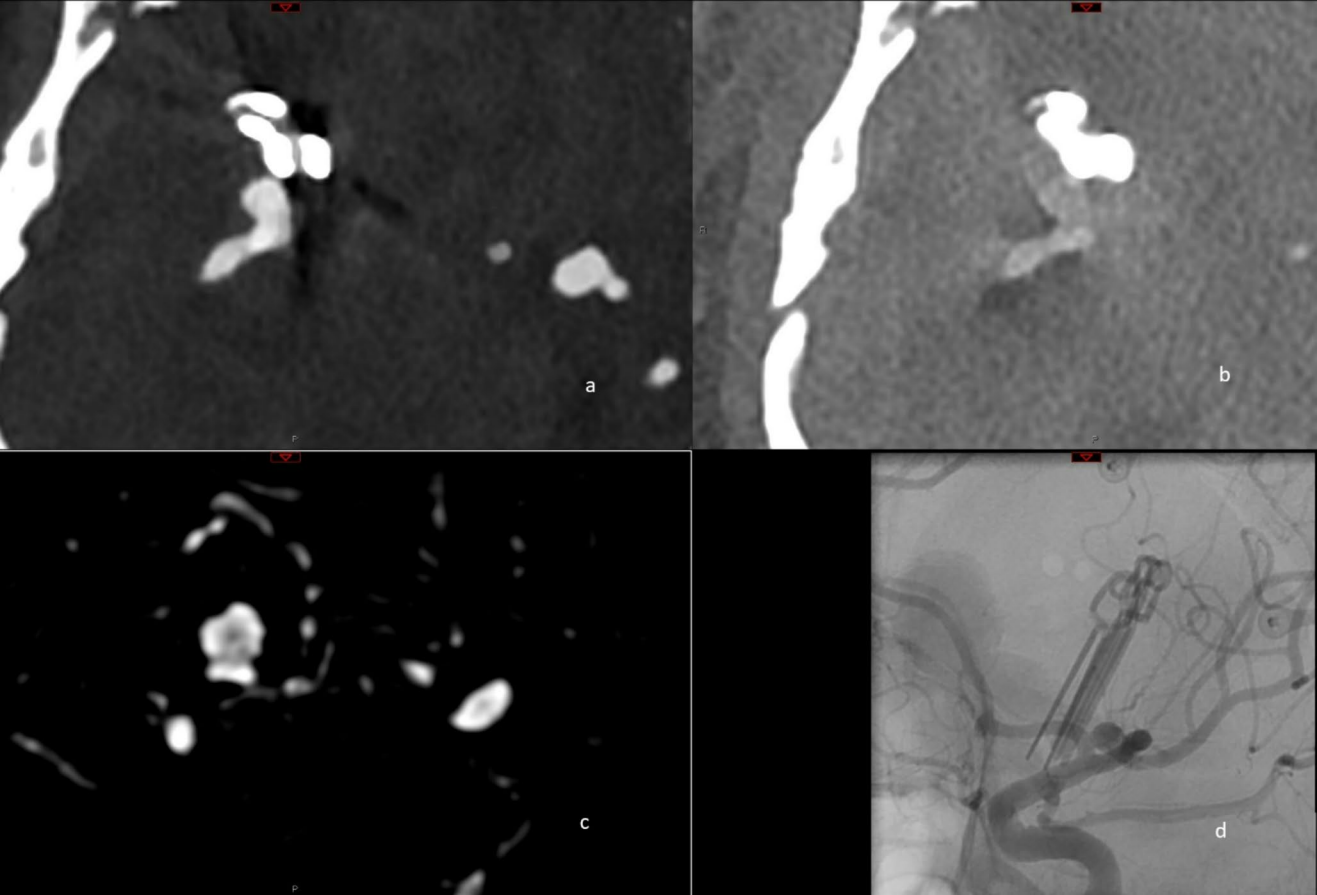
Illustration of the effect of the iMAR reconstruction on the vessel visibility in the immediate vicinity of the clip. A and B show an aneurysm remnant in the distal M1-segment of the right medial cerebral artery next to three clips. A is a VMI reconstruction with Hv56, B is a VMI reconstruction with Hv56 and iMAR. C and D show an axial reformation of a 3D DSA and a dedicated lateral 2D projections of the right medial cerebral artery. Abbreviations: Hv: Head vascular kernel, iMAR: iterative metal artefact reduction, keV: kiloelectron Volt, VMI: Virtual Monoenergetic Images

**Fig. 4:**
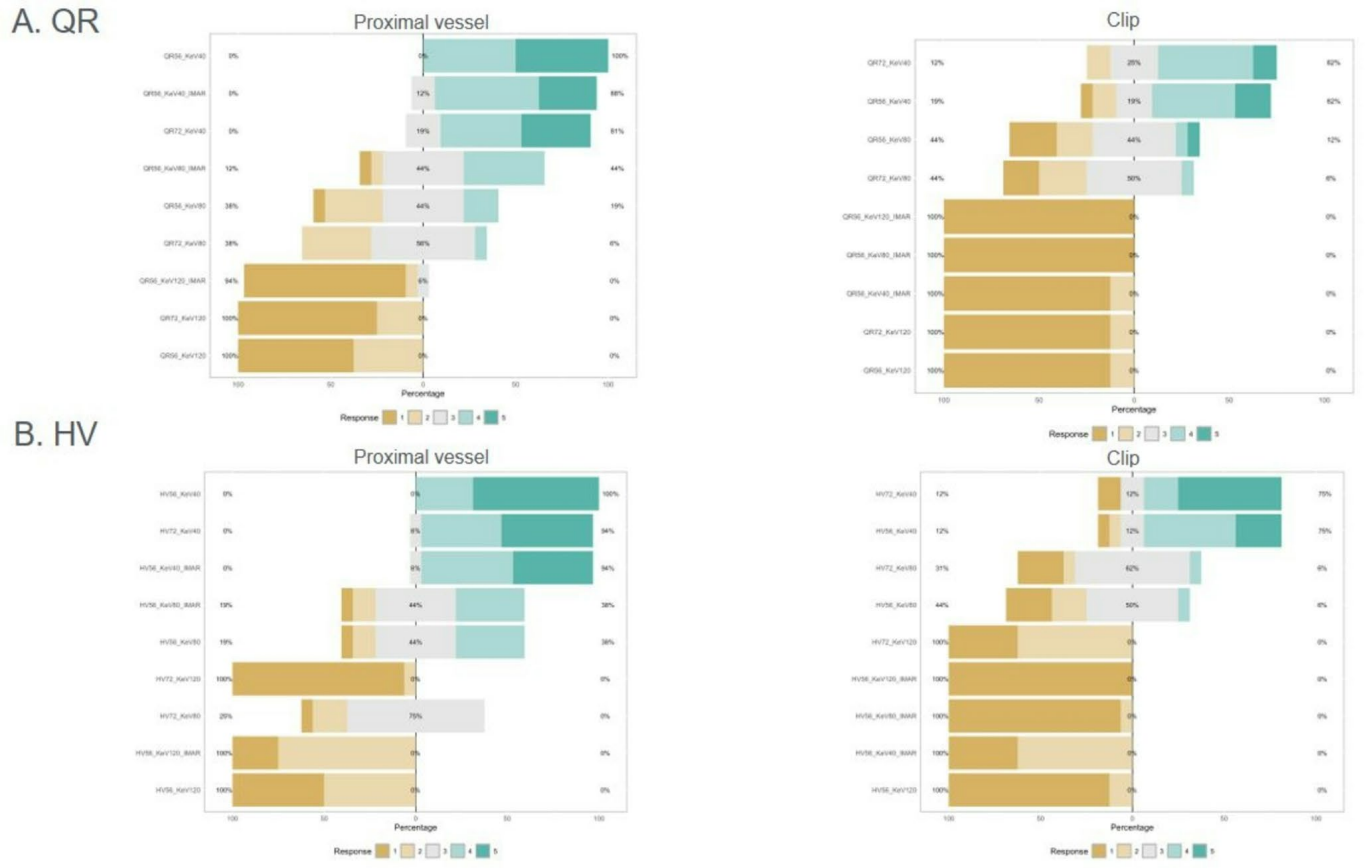
Qualitative image quality scores of the vessel segment at clip site for VMI with QrA) and Hv(B) with different kernels (Hv56 with an without iMAR, Qr56 with an without iMAR, Hv72 and Qr72) and keV levels (40, 80, 120). Stacked bar charts show pooled percentages of three raters for the vessel segment at clip site. Interpretation of scores: 5 = excellent image quality, 4 = good image quality, 3 = acceptable image quality, 2 = barely satisfactory image quality, 1 = unacceptable image quality. Abbreviations: Hv: Head vascular kernel, iMAR: iterative metal artefact reduction, keV: kiloelectron Volt, Qr: Quantitative kernel, VMI: Virtual Monoenergetic Images

At the clip or coil site, reconstructions with iMAR (Qr56 and Hv56) were rated significantly lower than those without iMAR, as visualized in Figs. 3 and 4.

For example, Hv56 with iMAR received a median score of 2, while Hv56 without iMAR had a median score of 4 (*p* = 0.002).

Similarly, PE and IOD reconstructed with iMAR were scored lower at the clip or coil site than images reconstructed without iMAR (Median score of iodine reconstructions vs. iMAR: 5 vs. 1 respectively, *p* < 0.001; and polyenergetic reconstructions without iMAR vs. with iMAR: 4 vs. 1, *p* < 0.001.)

Moderate agreement (κ = 0.59) was found between the two readers for qualitative ratings using the 5-point Likert-type scale. The internal consistency of the test, measured by Cronbach’s alpha, was excellent (α = 0.89).

Spectral reconstructions and PE were affected by coils in the vicinity of the vessel lumen (IOD-, PE, VMI-reconstructions: *p* = 0.005, *p* < 0.001, *p* < 0.001 respectively).

Detailed metrics of subjective and objective image quality are provided in the supplemental data.

#### Diagnostic Performance of PCD-CTA Compared with DSA

In 16/21 patients, PCD-CTA provided sufficient image quality to assess aneurysm occlusion and patency of the parent vessel. Whereas patients with clips presented in 15/17 cases sufficient image quality, only 1/4 patients with coils showed sufficient image quality. All aneurysm remnants were detected. In patients with clips, specificity of PCD-CTA was 70% (8/9) and in patients with coils 0%. Negative predictive value regarding the presence of an aneurysm remnant was 100% (9/9). Reconstructions with iMAR adversely influenced the evaluation: only 5/17 were of diagnostic quality at the clip site, as visualized in Fig. 3.

All readers observed a 100% negative predictive value for aneurysm occlusion. Inter observer agreement for diagnostic accuracy was excellent. (κ = 0.81, *p* = 0.002) Inter observer agreement for image quality was moderate (κ = 0.59, *p* < 0.001).

#### Assessment of radiation dose

The mean dose-length product was 113 mGycm ± 23, and the dose-area products was 1539 µGycm^2^ ± 648.

## Discussion

This study offers an in vivo comparison of Photon Counting Detector CT Angiography with DSA for evaluating intracranial aneurysms after microsurgical clipping or endovascular coil-embolization. Reconstructions using smoother kernels and lower keV levels demonstrated superior SNR and CNR outperforming those with harder kernels and higher keV levels. This result seems to be expected due to the physical principles, but a more significant artifact reduction was described at a higher keV level in an experimental setting [7]. Pallasch et al. reported that iMAR for photon-counting CT is an adapted solution of a previously introduced version developed for conventional dual-energy CT. With appropriate settings the iMAR algorithm does indeed reduce a large portion of metal artifacts produced by intracranial clips and coils but unfortunately renders the adjacent parent vessel invisible [18]. 

For this study, only the assessability of the parent vessel and the presence of an aneurysm remnant were relevant and not the overall artifact reduction, which might explain the discrepancy.

Iodine, polyenergetic, and virtual monoenergetic imaging reconstructions with the Hv56 kernel showed comparable performance at the clip or coil sites.

However, the application of iMAR negatively impacted image quality with 5/7 aneurysm remnants not being visible as visualized in Fig. 3. These findings diverge from the promising experimental data on Photon-Counting CT and the results of flat-detector panel CTA with iMAR [7, 19]. 

They align with previous studies indicating that iMAR can compromise contrast in vessels adjacent to coil packages and clips in CTA images [20, 21]. 

Fitsiori et al. found the presence of a halo like signal loss around coil packages. As far as these observations are comparable, we indeed noticed a similar phenomenon, that led to signal loss in the parent vessel next to the clips or coils [21]. Nonetheless, the artefacts in our patient collective seem less pronounced.

Alternative metal artifact reduction techniques, such as single-energy metal artifact reduction (SEMAR) in ultra-high-resolution CTA with different CT scanners, have been shown to reduce artifacts in the proximity of clips and coils.

However, the effectiveness of these methods was not correlated with other imaging modalities like DSA or MRI in these studies and no comparison to iMAR was performed [22–24]. 

Overall iMAR decreased the artifact burden, however iMAR could not sufficiently identify the most critical structure, namely the parent vessel of the aneurysm. Consequently, not only were the artifacts suppressed but also the vessel segment next to the coils. Of note also aneurysm remnants, that were not sufficiently occluded by coils/clips and could be visualized with CT-Angiography without iMAR reconstructions were also not visible in reconstructions with iMAR.

The extent of clip-related artifacts appeared to depend on the type of clips used. Despite this, variability in vessel evaluation persisted among patients with Yasargil^®^ clips, potentially due to factors such as the shape or number of clips, the clip-gantry angle, or the aneurysm’s location. Similarly, the assessment of vessels at coil sites was highly influenced by the coil-vessel angle and the aneurysm’s location. Artifacts were more pronounced for bigger aneurysms, i.e. more coils, but the artifacts hindered sufficient parent vessel visualization for all aneurysm sizes analyzed in this study.

### Limitations

This study has several limitations. First, the retrospective design and the small sample size limit the generalizability of our findings. However, we see the study’s value in its thorough analysis of the technical considerations that have to be made if PCD-CT is used for evaluating patients after coil-embolization or clipping. Image post processing offers many possibilities in PCD-CT imaging and improves diagnostic image quality. Additionally, radiologists evaluated the reconstructions in a standardized way, i.e. images were not randomly presented, which could introduce bias in image evaluation. Second, the study’s findings are specific to the metal artifact reduction (MAR) algorithm of a single vendor, meaning that these results may not be applicable to other MAR techniques from different vendors. Further research is necessary to evaluate and compare the performance of various MAR algorithms across different platforms. Lastly, in cases where PCT-CTA demonstrated sufficient diagnostic quality, postoperative DSA was not performed, resulting in 11 out of 21 patients in the study’s time window after microsurgical clipping not undergoing DSA.

## Conclusion

In this study, Photon-Counting Detector CT-Angiography (PCD-CTA) comprising ultra-high-resolution imaging demonstrated adequate diagnostic value in most patients with intracranial aneurysms after microsurgical clipping when implementing 40 keV virtual monoenergetic imaging. For patients after coil-embolization, PCD-CT did not reach diagnostic imaging quality. For both patient groups, iMAR reduced artifacts, but let to a significantly decreased visibility of the aneurysm’s adjacent parent vessel instead.

## Electronic supplementary material

Below is the link to the electronic supplementary material.

Supplementary Material 1

Supplementary Material 2

## Data Availability

Data are available upon reasonable request, but access is restricted due to legal and ethical reasons.

## Abbreviations

PCD-CTA: Photon-counting detector CT-Angiography
DSA: Digital subtraction angiography
UHR: Ultra-high-resolution
IOD: iodine
VMI: virtual monoenergetic imaging
PL: pure lumen
SNR: Signal-to noise ratio
CNR: Contrast-to noise ratio
keV: Kiloelectron volt
Qr: Quantitve, kernel type
Hv: Head vessel kernel type
