# Supplementary material for: Photon-counting CT-angiography in comparison to digital subtraction angiography for assessing intracranial aneurysms after coiling or clipping
Source: Neuroradiology. 2025 Jun 2;67(8):2021–30. doi: 10.1007/s00234-025-03650-w (PMC12494643; doi:10.1007/s00234-025-03650-w)
Supplement: Supplementary file 1 — Supplementary Material 1 [file 234_2025_3650_MOESM1_ESM.docx]

Table 1 Virtual monoenergetic reconstructions for Qr56 (iMAR) and Qr 72 Kernel for keV Level of 40. Quantitative image quality measurements of the PCD-CT. All values are reported as means and SD (Qr: quantitative kernel, CNR: Contrast-to-noise Ratio, IQR: Interquartile rande, iMAR: iterative metal artifact reconstruction, SD: standard deviation, SNR: signal- to-noise ratio)

|  | Qr 56 | Qr 56 IMAR | Qr 72 | p |
| --- | --- | --- | --- | --- |
| Lumen of proximal artery | | | | |
| SNR | 30,3 (10,2) | 27,8 +15 | 18,0 (7,3) | <0.001 |
| CNR | 38,8 (14) | 28,1 (16) | 22,7 (7,3) | <0.001 |
| Lumen at clip site | | | | |
| SNR | 15,9 (12,3)* | 8,5 (15,6)* | 12,8 +9 | .011 |
| CNR | 35,9 +19 | 13,9 + 13,5 | 19,8 + 11,3 | <0.001 |

All values are reported are means, medians are marked with a * ( CNR: Contrast-to-noise Ratio, IQR: Interquartile rande, iMAR: iterative metal artifact reconstruction, Qr: Quantitative kernel, SD: standard deviation, SNR: signal- to-noise ratio)

Table 1a Pairwise comparison for SNR in proximal vessel and clip for VMI (Qr)

|  | Qr 56 IMAR -QR 72 | Qr 56 -QR 72 | Qr 56 IMAR -QR 56 |
| --- | --- | --- | --- |
| Proximal vessel | 0.081 | <0.001 | 0,022 |
| Vessel at clip/coil site | .246 | .008 | .618 |

All values are reported are p-values

Table 1b Pairwise comparison for CNR in proximal vessel and clip for VMI (Qr)

|  | Qr 56 IMAR -QR 72 | Qr 56 -QR 72 | Qr 56 iMAR -Qr 56 |
| --- | --- | --- | --- |
| Proximal vessel | 0,034 | <0.001 | 0.002 |
| Vessel at clip/coil site | .464 | <0.001 | .003 |

All values are reported are p-values

Table 2 Virtual monoenergetic reconstructions for Qr56 and 72 kernel at three different keV Level Quantitative image quality measurements of the PCD-CT. All values are reported as means and SD (Qr: Quantitative kernel, CNR: Contrast-to-noise Ratio, IQR: Interquartile rande, keV: kiloelectron Volt, SD: standard deviation, SNR: signal- to-noise ratio)

|  | 40 keV | 80 keV | 120 keV | p |
| --- | --- | --- | --- | --- |
| Lumen of proximal artery | | | | |
| SNR | 23,7 (16,7)* | 9,9 (6,1)* | 5,9 (3,6)* | <0.001 |
| CNR | 33,3 (15,5)* | 12,7 (8,4)* | 6,7 (3,4)* | <0.001 |
| Lumen at clip site | | | | |
| SNR | 12,5 (15,9) | 6,7 +3,9 | 4,8 (2,8) | <0.001 |
| CNR | 23,2 +17,6 | 11,6 (10,5) | 6,2 (7,5) | <0.001 |

2a Pairwise comparison for SNR in proximal vessel

|  | keV 120 – keV 80 | keV 120 – keV 40 | keV 80 – keV 40 |
| --- | --- | --- | --- |
| Proximal vessel | <0.001 | <0.001 | <0.001 |
| Vessel at clip/coil site | <0.001 | <0.001 | <0.001 |

All values are reported are p-values

2b Pairwise comparison for CNR in proximal vessel

|  | keV 120 – keV 80 | keV 120 – keV 40 | keV 80 – keV 40 |
| --- | --- | --- | --- |
| Proximal vessel | <0.001 | <0.001 | <0.001 |
| Vessel at clip/coil site | <0.001 | <0.001 | 0.024 |

All values are reported are p-values

Table 3 Virtual monoenergetic reconstructions for Hv56 (iMAR) and Hv 72 Kernel for keV Level of 40. Quantitative image quality measurements of the PCD-CT. All values are reported as means and SD (Hv: Head vascular, CNR: Contrast-to-noise Ratio, IQR: Interquartile rande, iMAR: iterative metal artifact reconstructon, keV: kiloelectron Volt, SD: standard deviation, SNR: signal- to-noise ratio)

|  | Hv 56 | Hv 56 IMAR | Hv 72 | p |
| --- | --- | --- | --- | --- |
| Lumen of proximal artery | | | | |
| SNR | 32,4 +10,6 | 25,4 + 15,4 | 17,2 (7,1)* | <0.001 |
| CNR | 37,4 (17,3)* | 29,8 + 13,3 | 18,7 (10,8)* | <0.001 |
| Lumen at clip site | | | | |
| SNR | 17,6 + 10,9 | 11,7 (20,6)* | 13,3 + 7,1 | 0,026 |
| CNR | 36,9 + 17,9 | 12,9 + 15,6 | 20,7 + 9,7 | <0.001 |

All values are reported are means, medians are marked with a * ( CNR: Contrast-to-noise Ratio, IQR: Interquartile rande, SD: standard deviation, SNR: signal- to-noise ratio)

Table 3a Pairwise comparison for SNR in proximal vessel and clip for VMI (Hv)

|  | Hv 56 IMAR -Hv 72 | Hv 56 -Hv 72 | Hv 56 IMAR -Hv 56 |
| --- | --- | --- | --- |
| Proximal vessel | .119 | <0.001 | .119 |
| Vessel at clip/coil site | .008 | <0.001 | .034 |

All values are reported are p-values

Table 3b Pairwise comparison for CNR in proximal vessel and clip for VMI (Hv)

|  | Hv 56 IMAR -Hv 72 | Hv 56 -Hv 72 | Hv 56 IMAR -Hv 56 |
| --- | --- | --- | --- |
| Proximal vessel | .805 | .022 | .342 |
| Vessel at clip/coil site | .246 | <0.001 | 0.001 |

All values are reported are p-values

Table 4 Virtual monoenergetic reconstructions for Hv56 and 72 kernel at three different keV Level (. Quantitative image quality measurements of the PCD-CT. All values are reported as means and SD (Hv: head vascular, CNR: Contrast-to-noise Ratio, IQR: Interquartile rande, keV: kiloelectron Volt, SD: standard deviation, SNR: signal- to-noise ratio)

|  | 40 keV | 80 keV | 120 keV | p |
| --- | --- | --- | --- | --- |
| Lumen of proximal artery | | | | |
| SNR | 24,9 + 13,4 | 10,6 + 14,3 | 5,8 (3,9) * | <0.001 |
| CNR | 30,6 + 14,2 | 13,8 + 6,4 | 6,6 + 2,9 | <0.001 |
| Lumen at clip site | | | | |
| SNR | 13,6 (15,2)* | 6,8 (6,1)* | 4,9 (4,1) * | <0.001 |
| CNR | 23,5 + 17,8 | 11,6 (8,4)* | 8,9 (6,0)* | <0.001 |

4a Pairwise comparison for SNR in proximal vessel and clip site

|  | keV 120 – keV 80 | keV 120 – keV 40 | keV 80 – keV 40 |
| --- | --- | --- | --- |
| Proximal vessel | <0.001 | <0.001 | <0.001 |
| Vessel at clip/coil site | <.004 | <0.001 | <0.001 |

All values are reported are p-values (Hv: Head vascular kernel, CNR: Contrast-to-noise Ratio, IQR: Interquartile rande, SD: standard deviation, SNR: signal- to-noise ratio)

4b Pairwise comparison for CNR in proximal vessel and clip site

|  | keV 120 – keV 80 | keV 120 – keV 40 | keV 80 – keV 40 |
| --- | --- | --- | --- |
| Proximal vessel | <0.001 | <0.001 | <0.001 |
| Vessel at clip/coil site | <0.001 | <0.001 | .010 |

All values are reported are p-values (Hv: Head vascular kernel, CNR: Contrast-to-noise Ratio, IQR: Interquartile rande, keV: kiloelectron Volt, SD: standard deviation, SNR: signal- to-noise ratio)

Table 5 Iodine reconstructions for Hv56 and Hv 72 Kernel for keV Level of 40. Quantitative image quality measurements of the PCD-CT. All values are reported as means and SD (Hv: head vascular, CNR: Contrast-to-noise Ratio, IQR: Interquartile rande, keV: kiloelectron Volt, SD: standard deviation, SNR: signal- to-noise ratio)

|  | Hv 56 | Hv 56 iMAR | Hv 72 | p |
| --- | --- | --- | --- | --- |
| Lumen of proximal artery | | | | |
| SNR | 14,6 + 2,8 | 10,9 (7,6)* | 8,5 + 2,7 | <0.001 |
| CNR | 231,7 + 58,7 | 158 + 113 | 156,3 + 67 | 0.02 |
| Lumen at clip site | | | | |
| SNR | 7,8 (8,9)* | 2,7 (5,9)* | 4,8 (7,7)* | <0.001 |
| CNR | 195,5 (158) | 49,9 +58,9 | 123 + 102 | <0.001 |

All values are reported are means, medians are marked with a * ( CNR: Contrast-to-noise Ratio, Hv: Head vascular kernel, IQR: Interquartile range, iMAR: iterative metall artifact reduction, SD: standard deviation, SNR: signal- to-noise ratio)

Table 5a Pairwise comparison for SNR in proximal vessel and clip for Iodine (Hv)

|  | Hv 56 IMAR -Hv 72 | Hv 56 -Hv 72 | Hv 56 IMAR -Hv 56 |
| --- | --- | --- | --- |
| Proximal vessel | 0.034 | <0.001 | .342 |
| Vessel at clip/coil site | <0.001 | <0.001 | 1 |

All values are reported are p-values (Hv: Head vascular kernel, CNR: Contrast-to-noise Ratio, IQR: Interquartile rande, SD: standard deviation, SNR: signal- to-noise ratio)

Table 5b Pairwise comparison for CNR in proximal vessel and clip for VMI (QR)

|  | Hv 56 IMAR -Hv 72 | Hv 56 -Hv 72 | Hv 56 IMAR -Hv 56 |
| --- | --- | --- | --- |
| Proximal vessel | <0.001 | <0.001 | 1 |
| Vessel at clip/coil site | 0.034 | .034 | <0.001 |

All values are reported are p-values (Hv: Head vascular kernel, CNR: Contrast-to-noise Ratio, IQR: Interquartile rande, SD: standard deviation, SNR: signal- to-noise ratio)

Table 6 Polyenergetic reconstructions for Hv56 (Imar) and Hv 72 Kernel for keV Level of 40. Quantitative image quality measurements of the PCD-CT. All values are reported as means and SD (Hv: head vascular, CNR: Contrast-to-noise Ratio, IQR: Interquartile rande, keV: kiloelectron Volt, SD: standard deviation, SNR: signal- to-noise ratio)

|  | Hv 56 | Hv 56 iMAR | Hv 72 | p |
| --- | --- | --- | --- | --- |
| Lumen of proximal artery | | | | |
| SNR | 13,2 (14)* | 12,2 (7,8)* | 10,4 (7,3)* | <0.001 |
| CNR | 17,6 (3,1)* | 15,1 (5,3)* | 12,7 (5,5)* | <0.001 |
| Lumen at clip site | | | | |
| SNR | 9,8 + 6 | 5,3 (6,2)* | 7,4 (7,1)* | <0.001 |
| CNR | 20,7 + 9 | 7,4 + 4,3 | 15,4 + 3,4 | <0.001 |

All values are reported are means, medians are marked with a * ( CNR: Contrast-to-noise Ratio, IQR: Interquartile rande, iMAR: iterative metal artifact reconstruction, SD: standard deviation, SNR: signal- to-noise ratio)

Table 6a Pairwise comparison for SNR in proximal vessel and clip for Iodine(Hv)

|  | Hv 56 IMAR -Hv 72 | Hv 56 -QR 72 | Hv 56 IMAR -Hv 56 |
| --- | --- | --- | --- |
| Proximal vessel | 0.034 | <0.001 | 0.034 |
| Vessel at clip/coil site | .342 | <0.001 | 0.033 |

All values are reported are p-values (Hv: Head vascular kernel, CNR: Contrast-to-noise Ratio, IQR: Interquartile rande, SD: standard deviation, SNR: signal- to-noise ratio)

Table 6b Pairwise comparison for CNR in proximal vessel and clip for VMI (QR)

|  | Hv 56 IMAR -Hv 72 | Hv 56 -Hv 72 | Hv 56 IMAR -Hv 56 |
| --- | --- | --- | --- |
| Proximal vessel | 0.081 | <0.001 | 0.081 |
| Vessel at clip/coil site | 0.173 | <0.001 | 0.173 |

Note

All values are reported are p-values (Hv: Head vascular kernel, CNR: Contrast-to-noise Ratio, IQR: Interquartile rande, SD: standard deviation, SNR: signal- to-noise ratio)
