# Supplementary material for: Photon-counting CT-angiography in comparison to digital subtraction angiography for assessing intracranial aneurysms after coiling or clipping
Source: Neuroradiology. 2025 Jun 2;67(8):2021–30. doi: 10.1007/s00234-025-03650-w (PMC12494643; doi:10.1007/s00234-025-03650-w)
Supplement: Supplementary file 2 — Supplementary Material 2 [file 234_2025_3650_MOESM2_ESM.docx]

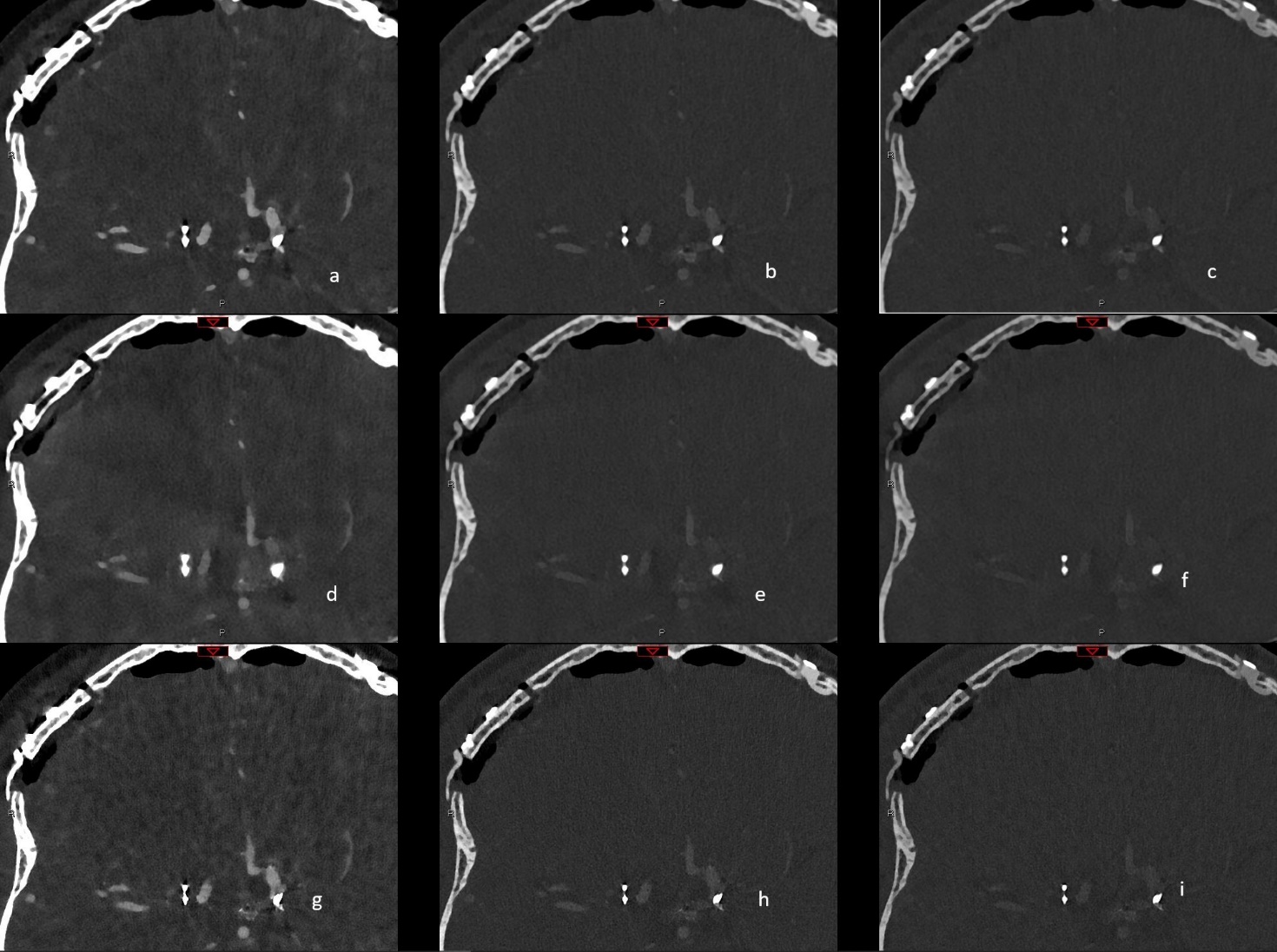


Fig.1 Illustration of reconstruction protocol for VMI with three different kernels and three keV levels. In the three rows, from left to right, the three keV levels (40, 80 and 120) are listed and in the three columns, from top to bottom Hv56 kernel without iMAR, Hv56 with iMAR and Hv 72. In detail: a = 40 keV, Hv 56; b = 80 keV, Hv 56; c = 120 keV, Hv 56; d = 40 keV, Hv56 with iMAR; e= 80 keV, Hv 56 with iMAR; f = 120 keV, Hv56 with iMAR; g = 40 keV, Hv 72; h = 80 keV; Hv72; i = 120 keV, Hv72

Abbreviations: Hv: Head vascular kernel, iMAR: iterative metal artefact reduction, keV: kiloelectron Volt, VMI: Virtual Monoenergetic Imaging


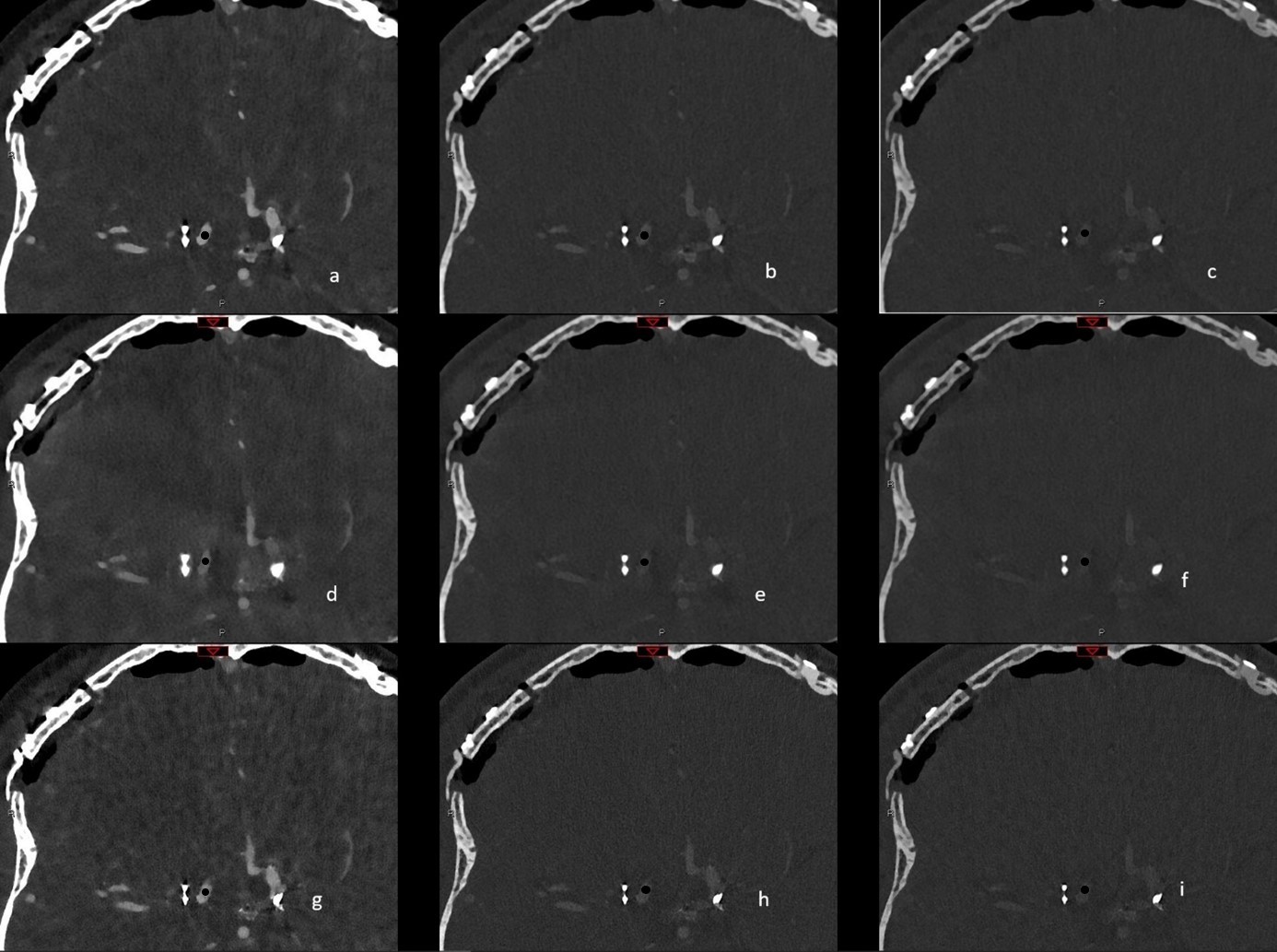


Fig.1.2 Same illustration of reconstruction protocol for VMI with exemplary ROIs inserted in the form of black dots.
